# Anti-convulsant efficacy of long-acting injectable cannabidiol formulation (IVL5005) in the pentylenetetrazol-induced convulsions, with pharmacokinetic characterization

**DOI:** 10.3389/fphar.2025.1692123

**Published:** 2025-10-23

**Authors:** Soyoung Youm, Joo Young Cha, Slgirim Lee, Young Dai Seo, Kun Hee Park, Aeri Song, Hyun-Je Park, Woo Chan Son, Juhee Kim

**Affiliations:** ^1^ Inventage Lab, Inc., Seongnam-si, Republic of Korea; ^2^ Department of Pathology, University of Ulsan College of Medicine, Asan Medical Center, Seoul, Republic of Korea; ^3^ Yuhan Care Co., Ltd, R&D Center, Yongin, Republic of Korea

**Keywords:** cannabidiol (CBD), CBD-loaded microspheres, long-acting injectable (LAI), epilepsy, pentylenetetrazole (PTZ) model, pharmacokinetics, anticonvulsant efficacy

## Abstract

**Introduction:**

Cannabidiol (CBD) has demonstrated therapeutic potential in neurological disorders, particularly epilepsy. Epidiolex®, an FDA-approved oral CBD solution, is indicated for rare epileptic disorders such as Lennox–Gastaut syndrome and Dravet syndrome. However, its clinical utility is limited by rapid metabolism, short duration of action, and low oral bioavailability.

**Methods:**

To address these limitations, we developed a long-acting injectable (LAI) formulation of CBD (IVL5005) using IVL-DrugFluidic® technology to achieve sustained and controlled drug release. CBD-loaded microspheres were manufactured and characterized by physicochemical analyses and *in vitro* release profiling. An *in vivo* pharmacokinetic study was conducted to evaluate systemic exposure following a single subcutaneous injection. The optimized formulation was selected for efficacy evaluation in a pentylenetetrazole (PTZ)-induced convulsion model.

**Results:**

All candidate formulations provided sustained systemic exposure for up to 4 weeks. The optimized IVL5005 formulation exhibited prolonged release with minimal initial burst. In the PTZ-induced convulsion model, IVL5005 demonstrated significant and durable anticonvulsant efficacy from a single dose, whereas the oral CBD solution produced only a transient effect. IVL5005 achieved a lower maximum plasma concentration compared with oral CBD solution, potentially reducing peak concentration-related adverse effects. No hepatic toxicity was observed with IVL5005, while liver changes were detected in the oral CBD group, likely due to extensive first-pass metabolism.

**Discussion:**

These results indicate that IVL5005 may overcome key limitations of oral CBD and support its further development as a long-acting therapeutic option for epilepsy.

## Introduction

Dravet syndrome (DS), or severe myoclonic epilepsy of infancy, is a rare epileptic disorder. It features various seizure types, with convulsive seizures being the most common (Anwar et al., 2019). DS incidence is approximately 1 in 20,000 births, with a prevalence of 3 per 100,000 individuals (EMA, 2019).

Lennox–Gastaut syndrome (LGS) is a severe epilepsy type starting before age 4, characterized by multiple seizure types such as tonic, atypical absence, atonic, myoclonic, and tonic-clonic seizures. Its causes are not well understood but may involve brain malformations, CNS infections, perinatal asphyxia, or severe head injuries. LGS incidence is approximately 1 in 40,000 births, with a prevalence of 15 per 100,000 individuals (EMA, 2019).

The long-term prognosis in children with LGS or DS varies significantly, but complete recovery with freedom from seizures is rare. Most children experience lasting cognitive impairments, and approximately half also face behavioral issues including hyperactivity, aggressive behavior, and social problems (Marchese et al., 2021). Furthermore, LGS patients continue to experience drug-resistant epilepsy, affecting over 90% (Goldsmith et al., 2000) of individuals.

Cannabidiol (CBD) has shown therapeutic potential as an anticonvulsant. It reduces neuroinflammation via NF- κB inhibition and lowers cytokine production (Kozela et al., 2010), with strong antioxidant properties. Many studies confirm CBD’s anticonvulsant effect in seizure models, possibly by modulating intracellular calcium and reducing neuronal hyperexcitability (Arzimanoglou et al., 2020; Consroe and Wolkin, 1977; Perucca, 2017). In clinical settings, CBD has been approved for refractory rare epilepsies such as Lennox–Gastaut syndrome (LGS) and Dravet syndrome (DS).

Epidiolex^®^, an oral CBD solution approved in 2018, treats seizures in LGS and DS. Clinical studies showed significant reductions in seizure frequency (Devinsky et al., 2017; Thiele et al., 2019; Perucca, 2017). However, CBD’s extensive liver and gut metabolism necessitate twice-daily dosing, which can be challenging for young patients. This highlights the need for drug delivery systems enabling consistent, convenient dosing.

Recent studies have explored various injectable formulations of CBD and THC aimed at providing prolonged therapeutic effect. For instance, an *in situ* forming implant (ISFI) formulation of CBD has been developed for the treatment of triple-negative breast cancer (TNBC), demonstrating promising results in slowing tumor progression (Lozza et al., 2025). Additionally, a subcutaneous liposomal CBD formulation has shown quantifiable plasma concentrations for up to 28 days in canine models, suggesting potential for chronic pain management (Shilo-Benjamini et al., 2022). These advancements highlight the growing interest in long-acting cannabinoid therapies. However, challenges remain in optimizing pharmacokinetic profiles and ensuring safety and efficacy.

Microsphere drug delivery systems have traditionally been fabricated using emulsion–solvent extraction/evaporation or spray-drying methods. While these approaches are widely applied, they often result in broad and non-uniform particle size distributions, complicating reproducibility of drug loading and lead to variability in drug release kinetics (Kim and Pack, 2006; José Carlos De La Vega., 2013). Because particle size directly influences release profiles, achieving a narrow and uniform size distribution is critical for consistent performance. In contrast, microfluidic-based methods enable precise control over droplet generation and microsphere formation by exploiting predictable fluid dynamics in microchannels (Xia and Pack, 2014; Rezvantalab and Mostafa, 2019). This approach produces highly monodispersed microspheres with reproducible drug incorporation and release behavior. However, large-scale production of microspheres using microfluidic systems can be challenging owing to their micro-scale size (Hu X et al., 2025). To address this, the IVL-DrugFluidic^®^ technology uses parallelized microfluidic chips for efficient production and precise control of size, morphology, and nanostructure (Chan et al., 2023).

We aimed to develop and assess a novel long-acting injectable (LAI) formulation of CBD, IVL5005, using IVL-DrugFluidic^®^ technology. CBD-loaded microspheres were formulated and evaluated in animal models to achieve sustained drug release and correlated anticonvulsant efficacy from a single injection.

## Materials and methods

### Materials

CBD-loaded microspheres were produced using CBD extracted from cultivated hemp, supplied by Yuhan Care R&D Center, with a purity greater than 99% according to the supplier’s certificate of analysis. PLGA copolymers with different ratios were used, including PLGA 5050A (acid-terminated 50/50 DL-lactide/glycolide), PLGA 7525A (acid-terminated 75/25 DL-lactide/glycolide), and PLA (poly (DL-lactide)). These materials were purchased from Purac^®^ Asia Pacific Pte. Ltd. The suspending vehicle for IVL5005 was a mixture of D-mannitol, sodium carboxymethylcellulose, and water for injection, and the same vehicle was administered to the vehicle control group. For intraperitoneal and oral administration, CBD was dissolved in medium-chain triglyceride (MCT) oil, and the resulting solution was used without further dilution.

### Formulation preparation

Microspheres containing CBD were fabricated using IVL-DrugFluidic^®^ technology, which employs a microfluidic system to co-inject biodegradable polymer and aqueous solutions into microchannels (Supplementary Figure S1). This approach allowed for highly controlled droplet formation, resulting in uniform and monodispersed spheres generated through the repulsive interaction between the two phases within the channels.

### Characterization of CBD-loaded microspheres

**FIGURE 1:**
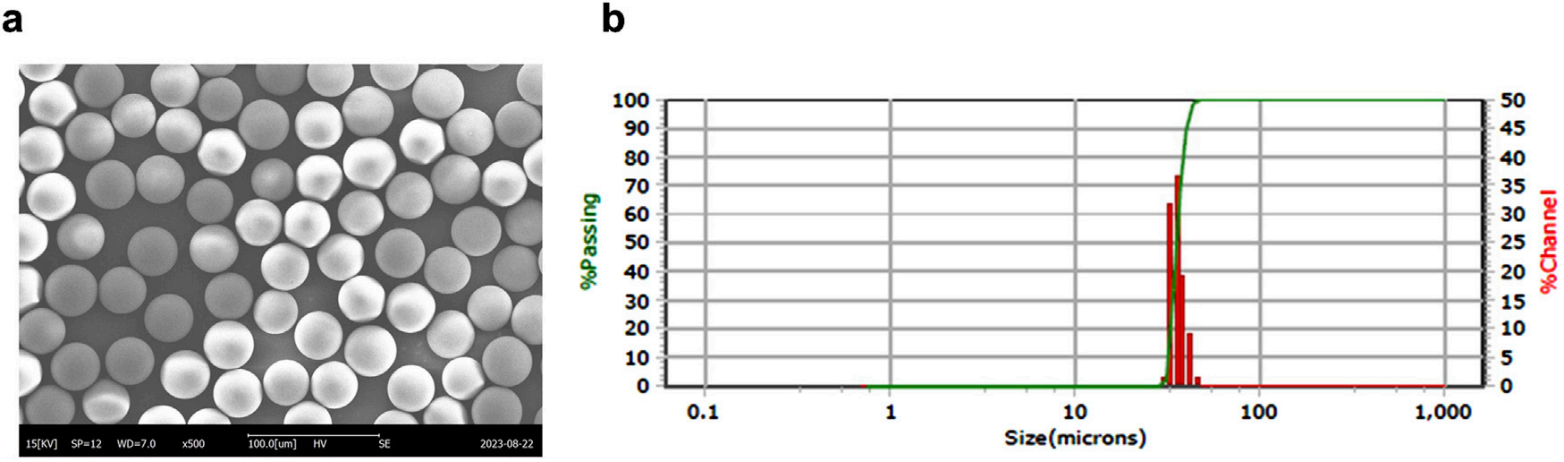
Manufacturing and characterization of CBD-loaded microspheres. **(a)** Representative SEM image of a CBD-loaded microsphere. **(b)** Particle size distribution of the microspheres. CBD, cannabidiol; SEM, scanning electron microscopy.

Microsphere morphology was observed using scanning electron microscopy (SEM, SNE-3000M, SEC Co., Ltd., Suwon, Korea), and size distribution was measured using a laser particle size analyzer (PSA, S3550, Microtrac Inc., Montgomeryville, PA, United States) (Figure 1).

Encapsulation efficiency are calculated as follows (Zhang et al., 2021) (Table 1):

$$EE\;\left(\%\right)=\frac{Actual\;amount\;of\;CBD\;in\;formulation}{Theoretical\;amount\;of\;CBD\;used\;for\;formulation}\times 100$$

**TABLE 1:** Formulation properties of CBD-loaded microspheres.

Test no.	D10 (μm)	D90 (μm)	Mean (μm)	CV (%)	Encapsulation efficiency (%)	Drug loading capacity (%)	Residual solvent (ppm)
#1	36.88	50.03	42.74	12.42	101.66	50.58	111.52
#2	32.45	45.93	37.95	13.64	104.00	20.76	59.72
#3	31.68	52.57	39.45	20.12	99.61	9.05	N.D
#4	32.84	47.50	38.92	14.26	98.72	32.80	N.D
#5	31.85	39.84	34.92	8.67	94.19	23.49	N.D
#6	32.83	43.71	38.05	11.56	101.59	20.28	N.D
#7	32.62	43.51	37.56	11.71	104.02	20.76	N.D
#8	33.10	44.67	38.64	11.80	98.33	9.82	N.D
#9	33.67	54.29	41.30	18.74	101.97	20.35	N.D

CBD, cannabidiol; CV, coefficient of variation; ppm, parts per million; D10, the diameter at which 10% of the particles have smaller diameters; D90, the diameter at which 90% of the particles have smaller diameters; N.D, not detected.

Drug loading capacity are calculated as follows (Table 1): Mass of drug in microspheres/Mass of microspheres) × 100.

Coefficient of variation (CV) for microsphere size was calculated as follows (Table 1): Standard deviation of microsphere diameters/Mean microsphere diameter X 100. The CV provides a measure of particle size uniformity, with lower values indicating higher homogeneity.

The size and size distribution of the microspheres are characterized using the median diameter and span value. Residual solvent levels in the microspheres were analyzed using gas chromatography (GC; YL6500). The analytical method was validated for limit of detection (LOD), limit of quantitation (LOQ), specificity, linearity, accuracy, precision, and system suitability. The LOD was defined as S/N ≥ 3 (for dichloromethane, S/N = 3.5, corresponding to 1.5 μg/mL) and the LOQ as S/N ≥ 10 (for dichloromethane, S/N = 59.2, corresponding to 3.0 μg/mL). Residual solvent levels below the LOD were reported as “not detected (ND)”.

### *In vitro* drug release assay

*In vitro* drug release testing was performed to evaluate the initial burst profile and formulation optimization. Because in vitro–in vivo correlation is often limited, *in vivo* PK studies in rats and dogs were relied upon primarily to evaluate formulation performance. The *in vitro* assay was therefore used mainly for early detection of excessive initial release and for establishing quality standards. For rapid comparison and quality evaluation of formulations, accelerated release testing was employed rather than long-term release testing. This approach was based on USP General Chapter <1001> (United States Pharmacopeia, 2022), using the incubation jar method at 50 °C and 120 rpm to achieve faster release conditions. Sampling points at 2, 18, and 72 h were selected based on approximate release levels of ≤30%, 50%–70%, and >80%, respectively. Formulations showing >30% release at 2 h were considered to exhibit an initial burst and were adjusted by increasing polymer content or using polymers with higher lactic acid ratios. Formulations meeting the ≤30% release criterion at 2 h were selected as candidates for subsequent *in vivo* PK studies.

Microspheres (15 mg) were dissolved in 100 mL PBS containing Tween 20 (0.05%), sodium azide (0.02%), and sodium ascorbate (0.0625%). Samples were collected at 2, 18, 72 h and analyzed using HPLC with UV detection at 220 nm (e2695 and 2998 PDA, Waters Corp., Milford, MA, United States).

### Pharmacokinetic studies

Pharmacokinetic (PK) studies were conducted in rats and dogs to evaluate systemic exposure of CBD from different formulations. In rats, five distinct CBD-loaded microsphere formulations were administered via a single subcutaneous injection at 9 mg/rat to five male Sprague-Dawley (SD) rats per formulation. In a comparative study, the long-acting injectable formulation was administered alongside an oral CBD solution at 60 mg/rat to fifteen male SD rats. In dogs, six male animals received a single subcutaneous injection of the selected long-acting formulation at 100 mg/dog. For rat studies, 500–1,000 μL blood samples were collected from the jugular vein at each time point, and plasma was separated and stored for analysis. For dog studies, approximately 1.5 mL of blood was collected via the cephalic vein, followed by centrifugation to isolate plasma for subsequent analysis. Blood samples were collected over a 4-week period, and plasma was separated and stored at −80 °C until analysis. Plasma CBD concentrations were quantified using a newly developed method employing an Acquity UPLC^®^ system (Waters, United States) coupled with a TQ5500 triple quadrupole mass spectrometer (SCIEX, United States).

### Animal efficacy study

#### Pentylenetetrazole-induced convulsions in rats

Pentylenetetrazole (PTZ; Sigma-Aldrich) was administered intraperitoneally at 37.5 mg/kg on days 1, 2, 3, 5, 7, 14, 21, and 28 to induce convulsion. Our protocol for PTZ administration involved daily administration of PTZ from day 1–3, every other day from day 5–7, and then weekly until day 28 (Singh et al., 2021). Although our protocol does not meet the criteria for a fully kindled state, our dosing schedule ensured adequate convulsion expression to evaluate the anticonvulsant effects of the investigational compound (Supplementary Figure S2). On day 1, rats received a single subcutaneous dose of the CBD-containing long-acting injectable (LAI; IVL5005) at either 30 mg/rat (low dose) or 60 mg/rat (high dose). Thirty minutes after dosing, PTZ was administered intraperitoneally, consistent with previous studies showing that CBD pretreatment 30 min prior to PTZ challenge produces sufficient absorption and reliable anticonvulsant effects (Vilela et al., 2017). The seveiry of seizure was evaluated for 30 min following PTZ injection. The CBD solution was administered either intraperitoneally to 10 rats or orally to 5 rats at 60 mg/rat on days 1 and 14. Clinical signs refers to general observations of the animal’s health and behavior, conducted in addtion to monitoring seizure activity. After each PTZ challenge, rats were returned to their home cages and observed twice daily (morning and afternoon) throughout 4-week study period to detect any abnormal signs, morbidity, or mortality. Oral administration of the CBD solution was performed using a 1 mL syringe equipped with a disposable oral zonde. The zonde was gently inserted into the rat’s mouth, and the solution was delivered slowly to ensure complete ingestion. Animals were briefly observed afterward to confirm full dosing and monitor for any signs of distress. On day 28, surviving animals were sacrificed, and brain tissues were fixed in 10% neutral buffered formalin. The study design is illustrated in Figure 3a. CBD was dissolved in medium-chain triglyceride (MCT) oil, and the resulting solution was used for both intraperitoneal and oral administration.

Seizure severity was assessed 30 min after each PTZ injection using the following scale, as described in previous study (Akyuz et al., 2020): 0, no response; 1, ear and facial twitching; 2, convulsive waves axially through the body; 3, myoclonic jerks and rearing; 4, clonic convulsions with the animal falling on its side; 5, repeated severe tonic-clonic convulsions; 6, death associated with seizure.

### Animal toxicity study

Nine male Sprague-Dawley (SD) rats were used per group for the oral CBD group (60 mg/rat/day) and the IVL5005 subcutaneous injection groups at 30 mg/rat and 60 mg/rat. The vehicle control group included three rats. Dose levels were selected based on the effective doses observed in the PTZ-induced convulsion study. Animals were sacrificed at the end of the 4-week study period, and histopathological analyses were performed on a full set of organs.

For pharmacokinetic analysis of CBD and its inactive metabolite 7-COOH-CBD (CBD-M), a separate set of six male SD rats received a single subcutaneous dose of IVL5005 at 8 mg/rat, slightly lower than the originally planned 9 mg/rat, due to limited availability of the drug formulation. This minor dose reduction is not expected to affect the assessment of parent drug and metabolite ratios, and it was sufficient to achieve measurable plasma concentrations for P analysis. Blood samples were collected at 16 time points (predose, 1 h, 2 h, 4 h, 8 h, 12 h, D2, D3, D4, D5, D6, D8, D11, D15, D22, D29) over 4 weeks to evaluate metabolite formation and its potential contribution to hepatic toxicity.

### Histopathological evaluation

#### Tissue preservation

Tissues from the efficacy and toxicity study were fixed in 10% neutral buffered formalin except for the testes, which were initially fixed using a modified Davidson’s fixative and then transferred to formalin.

### Hematoxylin and eosin (H&E) staining

Selected tissue sections (Supplementary Table S9) were fixed in 10% neutral buffered formalin, embedded in paraffin, and sectioned at 4–5 μm thickness. Sections were deparaffinized in xylene, rehydrated through graded ethanol, and stained sequentially with hematoxylin and eosin using a standard protocol. Stained slides were dehydrated, mounted, and examined under a light microscope (Olympus BX52 microscope). Histopathological evaluation focused on cellular morphology, necrosis, inflammation, and overall tissue architecture.

### Nissl staining

Brain sections from the PTZ efficacy study were prepared from the right hemisphere, fixed in 10% formalin, and trimmed in the coronal plane at bregma −2.5 to −3.8 to include the hippocampus. Sections were processed and embedded in paraffin, and four consecutive sections were prepared, with one section per slide stained with Nissl stain. The number of Nissl-stained cell bodies in the hippocampal CA1, CA3, and dentate gyrus (DG) regions was quantitatively analyzed at high-power fields (×400 magnification) following a previously described method (Sohn et al., 2019). Images were captured using an optical microscope (BX61, Olympus, Japan) and DP80 camera (Olympus, Japan), and quantitative analysis was performed using Image-Pro software (Media Cybernetics, United States).

### Immunohistochemical staining

Brain sections of the hippocampal region (4–5 μm thickness, formalin-fixed and paraffin-embedded) were deparaffinized in xylene and rehydrated through graded ethanol. Antigen retrieval was performed by immersing the sections in 0.01 M citrate buffer and heating in a pressure cooker to full pressure for 10 min. Nonspecific binding was prevented using a blocking reagent (Vector Laboratories, United States) for 30 min at room temperature. Sections were incubated overnight at 4 °C with anti-IBA1 antibody (rabbit polyclonal, ThermoFisher, United States) diluted 1:500 in PBS, followed by three washes with PBS. After primary antibody incubation, sections were incubated with a fluorescence-conjugated secondary antibody (goat anti-rabbit IgG, Alexa Fluor 488, Invitrogen, United States) for 1 h at room temperature in the dark. IBA1 is a marker of microglial activation (microgliosis), which is a pathological feature observed in seizures (Zhang et al., 2022; Santos and Fields, 2021).

Fluorescent images were captured using a fluorescence microscope at ×20 and ×40 magnifications. Quantitative analysis of IBA1-positive signals in the hippocampal region, which included the CA1, CA3, and DG areas was performed using Image-Pro software (Media Cybernetics, United States). For each section, regions of interest (ROI) of identical size were selected, and positive signal intensity was measured. The values were normalized relative to the control group (G1) and expressed as percentage change.

### Statistical analysis

All numerical data were analyzed using GraphPad Prism software (Version 8.0). The group differences were evaluated using one-way analysis of variance (ANOVA) followed by Fisher’s LSD test. Differences were considered statistically significant at p < 0.05 or at p < 0.01. All data are presented as mean ± standard error of the mean (SEM).

## Results

### CBD-loaded microsphere manufacturing and characterization

To establish optimal conditions for producing microspheres, various CBD-loaded microspheres were prepared using IVL-DrugFluidic^®^ (Supplementary Figure 1a). The interaction between the polymer solution and the aqueous phase within microchannels generated monodispersed droplets under controlled flow conditions. We analyzed the morphology and size distribution of microspheres using SEM, revealing smooth and perfectly spherical particles (Figure 1a). Notably, PLGA and PLA were selected as the matrix polymers because their degradation rate can be tuned by varying the lactic acid to glycolic acid ratio, thereby enabling control of CBD release kinetics and minimizing burst release. The size distribution was narrow and monodispersed, with median diameters ranging from 34.92 to 42.74 µm (Figure 1b; Table 1). The coefficient of variation (CV%) remained below 20%, reflecting a high level of uniformity. The drug encapsulation efficiency was consistently above 80%, and residual solvent levels were within the permissible limit of 600 ppm.

### *In vitro* drug release

**FIGURE 2:**
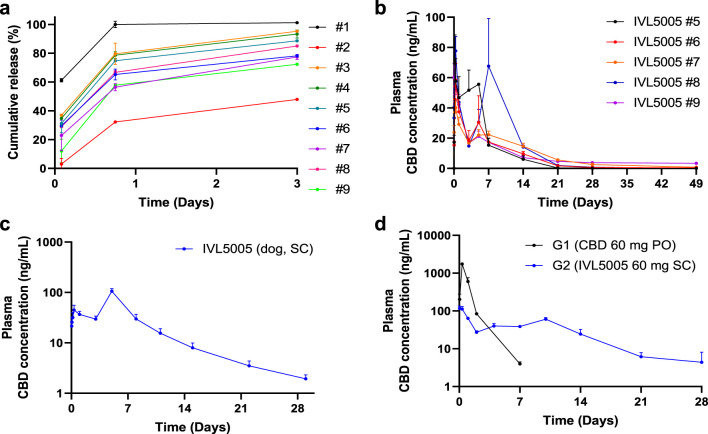
*In vitro* drug release and in vivo pharmacokinetic (PK) tests in rats. **(a)** *In vitro* drug release test. **(b)** *In vivo* rat PK of five CBD-containing long-acting injectable (LAI) microspheres (plasma, 5 rats/group). **(c)** *In vivo* dog PK of CBD LAI (plasma, 6 dogs/group). **(d)** *In vivo* rat PK comparison between CBD solution and CBD LAI (plasma, 15 rats/group). Values are expressed as mean ± standard error of the mean. CBD, cannabidiol; PK, pharmacokinetics.

Accelerated release testing revealed the release profiles of the CBD-loaded microspheres (Figure 2a). Test #1 released over 60% within 2 h and nearly 100% by 18 h, indicating insufficient sustained-release performance. In contrast, Test #2 exhibited a slower release (∼50% by 72 h), suggesting improved duration of action but limited early exposure. Tests #3 to #9 showed more balanced profiles, releasing 50%–80% by 18 h with incomplete release at 72 h. Among these, #3 and #4 showed relatively faster release kinetics. Based on these results, formulations #5 to #9 were selected for further evaluation due to their favorable sustained and balanced release characteristics.

### Pharmacokinetics of CBD-loaded microspheres in SD rats and beagle dogs

PK studies demonstrated sustained drug release over 4 weeks (Figure 2). In the rat PK study (Figure 2b), all five formulations, each containing 30 mg of CBD, exhibited Cmax values ranging from 41.31 to 102.53 ng/mL. The initial burst release, estimated by the ratio of AUClast and AUC0–24 h, was below 20% for all formulations. Tests #6, #7, and #9 showed relatively lower Cmax values (59.22, 41.31, and 68.96 ng/mL, respectively). Notably, Tests #7 and #9 maintained sustained release profiles, with plasma concentration of 0.6 and 2.5 ng/mL at 672 h, respectively. Test #7 was selected for further investigation due to its favorable polymer composition, which enables formulation at a lower injection volume. In the dog PK study with Test #7 (Figure 2c), a sustained plasma exposure was observed over a 4-week period, consistent with the results in rats. Test #7 was selected for subsequent studies, hereafter referred to as CBD-LAI (IVL5005) throughout the manuscript and figures to maintain consistent terminology.

### PK comparison between CBD solution and CBD-containing LAI

A comparison of PK profiles revealed marked differences between the oral CBD solution (PO) and subcutaneously administered IVL5005 (SC)¶. The Cmax of the IVL5005 group was significantly lower than that of the PO group, showing a 14.9-fold reduction (Table 2; Figure 2d). In the PO group, Cmax was reached at 8 h post-dose, followed by a rapid decline, with plasma concentrations falling below the limit of quantification by 92 h. In contrast, the IVL5005 group achieved Cmax at 1 h and demonstrated a sustained, controlled-release profile, with measurable plasma levels maintained for up to 672 h following single injection.

**TABLE 2:** *In vivo* rat PK parameters and comparison between CBD solution and CBD LAI.

Test no.	Dose mg/head	Dose (mg kg^-1^)	Dosing regimen	T_max_ (h)	C_max_ (ng mL^-1^)	AUCl_ast_ ng × h/mL	AUC_inf_ ng × h/mL
CBD LAI (#7)	60	200	Single SC injection	1	116.6	17,878.3	18,587.9
CBD solution	60	200	Single PO administration	8	1742.3	33,540.0	33,651.1

CBD, cannabidiol; LAI, long-acting injectable; PK, pharmacokinetics; SC, subcutaneous; PO, oral administration; T_max_, time to maximum concentration; C_max,_ maximum concentration; AUC_last_, area under the curve from time zero to last measurable concentration; AUC_inf_, area under the curve from time zero to infinity.

### CBD-containing LAI efficacy in the animal convulsion model

#### Mortality

**FIGURE 3:**
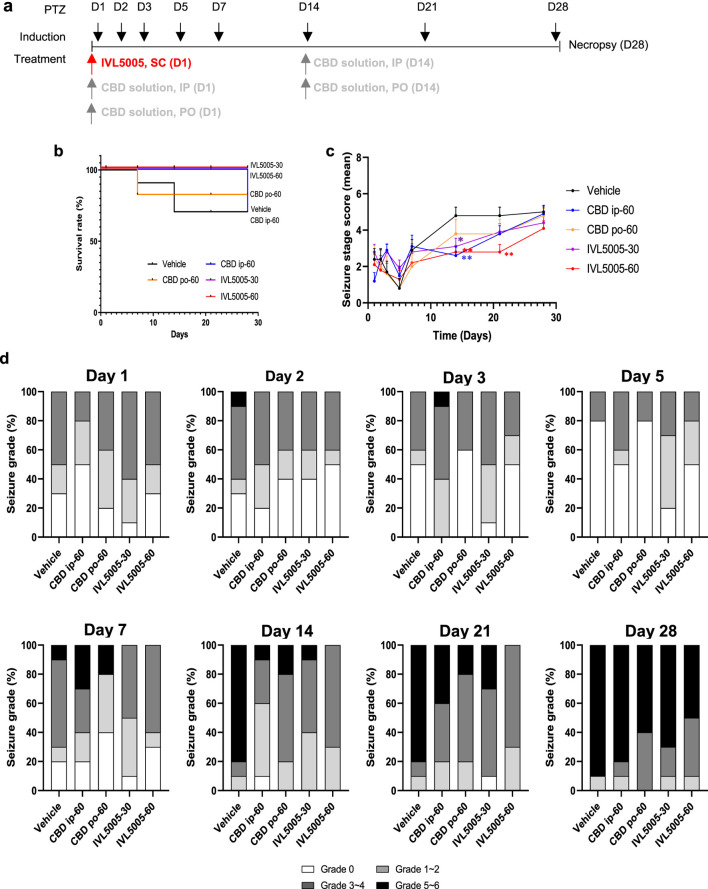
Efficacy of CBD in the rat seizure model. **(a)** Study design for the PTZ-induced rat seizure model. **(b)** Survival rate in the PTZ-induced rat seizure model. **(c)** Mean seviery of seizure in the PTZ-induced rat seizure model. **(d)** Distribution of seviery of seizure in the PTZ-induced rat seizure model. Values are expressed as mean ± standard error of the mean. *p < 0.05 vs. 18 Vehicle group. CBD, cannabidiol; PTZ, pentylenetetrazole. Number of animals: G1 (vehicle), n = 10; G2 (CBD IP), n = 10; G3 (CBD oral), n = 5; G4 (IVL5005 30 mg), n = 10; G5 (IVL5005 60 mg), n = 10.

In the vehicle control group, seizure-related deaths occurred progressively during the study, with the first death on day 7 and two additional deaths by day 14, resulting in a 70% survival rate. In the CBD IP group, three animals died on day 28, also yielding a 70% survival rate. In the CBD oral group, one of five animals died on day 7, resulting in an 80% survival rate. In contrast, no deaths were observed in either the low- and high-dose IVL5005 groups, corresponding to a 100% survival rate (Figure 3b).

#### Seizure score evaluation

PTZ-induced seizures were used to evaluate anticonvulsant efficacy (Figure 3c). In the vehicle group, seizure severity increased from day 14, reaching scores of 4.8–5.0 by day 28. IP injection of CBD on days 1 and 14 reduced seizure scores by 71% (day 1) and 60% (day 14). Oral CBD also showed a tendency to reduce seizure scores on day 14, but the effect was not statistically significant. A single dose of IVL5005 on day 1 produced reductions in seizure severity. Significant reductions were observed on day 14 for both low (IVL5005-30) and high (IVL5005-60) doses, and on day 21 for the high dose (IVL5005-60). Reductions at other time points, including day 28, were observed compared with vehicle, but did not reach statistical significance.

#### Seizure score distribution

Severity of seizure was assessed on a scale from grade 0 to grade 5, with grade 6 indicating seizure-induced death. Grades 1–2 denote mild seizures, grades 3–4 moderate seizures, and grades 5–6 severe seizures, close to death (Figure 3d).

By day 14, 80% of animals in the vehicle group exhibited severe seizures. In contrast, severe seizure incidence decreased to 10% with the second IP administration of CBD solution and 20% with the second oral administration, with the IP route showing greater efficacy. In the IVL5005 groups, severe seizures were reduced to 10% (low dose) and completely prevented (0%) in the high dose group.

On day 21, severe seizures were observed in 20%–40% of animals treated with the CBD solution, compared to 30% in the low-dose IVL5005 group and 0% in the high-dose group. Most high-dose animals exhibited only mild to moderate seizures.

By day 28, severe seizures persisted in 90% of vehicle-treated and 80% of CBD IP-treated animals. In the IVL5005 groups, although the proportion of moderate to severe seizures increased, the incidence of severe seizures remained lower, at 70% (low dose) and 50% (high dose).

### Hippocampal neuronal loss (Nissl staining)

**FIGURE 4:**
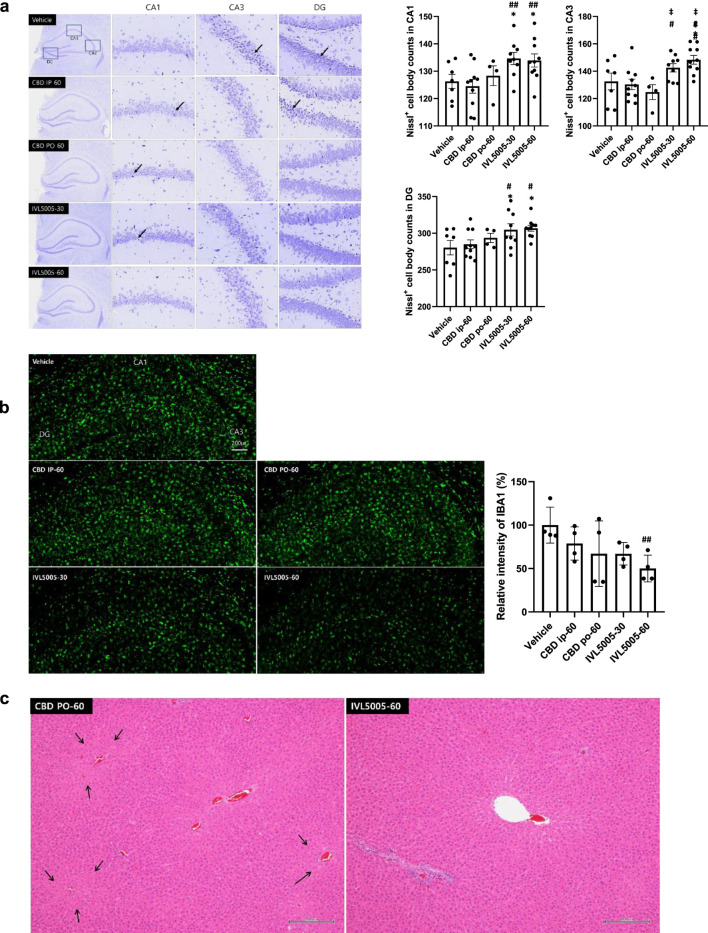
Histopathological evaluation. **(a)** Neuronal loss in the hippocampus (Nissl staining); dark-colored, shrunken neurons, indicative of degenerated neuronal cells (arrow). **(b)** Neuronal inflammation (immunostaining for IBA1). **(c)** Liver hypertrophy (arrow, H&E staining). Values are expressed as mean ± standard error of the mean. *p < 0.05 vs. Vehicle group, #p < 0.05 vs. CBD IP group, ##p < 0.01 vs. CBD IP group, ‡p < 0.05 vs. CBD PO group. CBD, cannabidiol; PK, pharmacokinetics. G1 (vehicle), n = 10; G2 (CBD IP), n = 10; G3 (CBD oral), n = 5; G4 (IVL5005 30 mg), n = 10; G5 (IVL5005 60 mg), n = 10.

Seizure induction resulted in neuronal damage, particularly in the hippocampus, a region highly sensitive to injury. Neuronal integrity was evaluated by Nissl staining (Figure 4a; Supplementary Table S3). The vehicle group exhibited numerous dark, shrunken neurons (indicated by arrows), indicative of degeneration, and showed the lowest number of Nissl-positive cells. The CBD IP and CBD PO groups demonstrated comparable neuronal loss. In contrast, the IVL5005 groups preserved a greater number of intact neurons, with the high-dose group showing marked improvement in comparison to the vehicle control. These findings correspond to the reductions in seizure severity observed on days 14 and 21, which were reflected at necropsy on day 28.

#### Neuronal inflammation (immunostaining for IBA1)

Microglial activation contributes to neuroinflammation and the progression of epilepsy (Drenthen et al., 2020). Neuronal inflammation was assessed by immunostaining for ionized calcium-binding adaptor molecule 1 (IBA1) (Figure 4b). The CBD IP and CBD PO groups showed a slight, non-significant decrease in IBA1 intensity compared to the vehicle control group. In contrast, high-dose IVL5005 group demonstrated a significant reduction in IBA1 expression, with a dose-dependent trend.

### CBD-containing LAI toxicity in normal SD rats

#### Histopathological analysis

Comprehensive organ histopathology revealed treatment-related changes in the liver and adrenal gland. In the oral CBD 60 mg group, centrilobular hepatocellular hypertrophy and increased adrenal vacuolation were observed (Table 3; Figure 4c). In contrast, no such changes were detected in the IVL5005 subcutaneous groups (30 and 60 mg). No abnormalities were observed in other organs, including the brain, adrenal gland, thyroid gland, kidney and spleen.

**TABLE 3:** Incidence and severity of the remarkable microscopic findings in rat toxicity study.

Group	Vehicle	CBD PO	IVL5005 SC	
Dose (mg/animal)	0	60	30	60
Route of administration*	SC	PO	SC	SC
Number of animals	3	9	9	9
Gland, Adrenal Examined	3	9	9	9
Vacuolation, cortex, zona fasciculata, increased, diffuse	—	5	—	—
Grade 1	—	2	—	—
Grade 2	—	3	—	—
Liver				
Examined	3	9	9	9
Hypertrophy, centrilobular, hepatocyte	—	4	—	—
Grade 1	—	4	—	—

CBD, cannabidiol.

#### CBD and CBD-M analysis

Toxicokinetic parameters of CBD and its primary inactive metabolite (7-COOH-CBD; CBD-M) are summarized in Table 4. Following a single subcutaneous administration of IVL5005, blood CBD concentration reached a Cmax of 19.3 ng/mL at 2 h, while CBD-M reached a Cmax 16.4 ng/mL at 12 h. The AUC_inf values were 8,936 ng*h/mL for CBD and 2,737 ng*h/mL for CBD-M, yielding a metabolite-to-parent ratio of 0.31.

**TABLE 4:** Toxicokinetic parameters of CBD and CBD-M following IVL5005 8 mg/head SC administration.

Analysis	C_max_ (ng/mL)	Tmax (h)	AUC_last_ hg × h/mL	AUC_inf_ ng × h/mL
CBD	19.3	2.0	4,099	8,936
CBD-M	16.4	12.0	2,199	2,737
CBD-M/CBD	0.85	-	0.54	0.31

CBD, cannabidiol; CBD-M, cannabidiol metabolite (7-COOH-CBD), c_max,_ maximum concentration; AUC_last_, area under the curve from time zero to last measurable concentration; AUC_inf_, area under the curve from time zero to infinity; SC, subcutaneous.

## Discussion

We developed and evaluated a novel LAI formulation of CBD, IVL5005, using IVL-DrugFluidic^®^ technology. IVL5005 achieved sustained systemic exposure to CBD for up to 4 weeks following single subcutaneous injection and significantly reduced seizure severity in a PTZ-induced convulsion model. This prolonged anticonvulsant efficacy, combined with lower peak plasma concentrations compared to oral CBD, suggests that IVL5005 may offer a promising strategy for improving seizure management through enhanced adherence and a reduced risk of adverse effects.

CBD’s anticonvulsant efficacy is well documented in preclinical and clinical studies. Consequently, the oral CBD solution Epidiolex^®^ was approved for LGS and DS. However, owing to its poor physicochemical properties, several strategies have been employed to enhance CBD bioavailability (O'Sullivan et al., 2024). Given CBD’s lipid solubility, numerous studies have explored its PK profiles with various carriers, such as coconut, sesame, and sunflower oils (Brookes et al., 2023; Schwotzer et al., 2023).

The IVL-Drug Fluidic^®^ technology enables controlled drug release by optimizing key factors like microsphere size, distribution, polymer type, and polymer-to-API ratio. The particles exhibited a narrow, monodispersed size distribution, crucial for consistent *in vivo* release. Small microspheres (Lin et al., 2018) can cause a higher initial drug burst and side effects, while larger ones may release slower and cause blockages. Thus, uniform, appropriately sized microparticles are essential for effective delivery.

In the rat PK study, oral CBD solution exhibited systemic exposure for approximately 1 week, with a peak plasma concentration at 8 h followed by a rapid decline. In contrast, the IVL5005, administered subcutaneously at the same dose, maintained systemic exposure for up to 4 weeks. Notably, the Cmax of the LAI formulation was 14.9-fold lower than that of the oral solution. A similar sustained exposure profile was also observed in non-rodent species, including beagle dogs.

Controlled release is a key consideration in LAI development, as an excessive initial burst can reduce therapeutic duration and increase the risk of toxicity (Yoo and Won, 2020). The IVL5005 formulations exhibited an initial burst release of 8.40%–13.23% on day 1, followed by sustained and stable release over 4 weeks. These results demonstrate a controlled, long-acting PK profile suitable for extended dosing intervals.

To confirm the efficacy of the IVL5005 compared to the CBD solution, anticonvulsant effects were evaluated using the PTZ-induced convulsion model in rat, in which repeated low-dose PTZ administration induces neuronal hyperexcitability primarily through GABA receptor antagonism Davoudi et al., 2013. Although CBD does not directly act on GABA receptors, it has been shown to effectively attenuate PTZ-induced neuronal hyperexcitability in this model, resulting in measurable anticonvulsant effects (EMA, 2019).

In our study the initial seizure score in the control group was 3.4 and gradually increased over time, reaching 4.8 by day 14, indicating full seizure induction, reflecting maximal seizure activity. To assess the efficacy of CBD under these conditions, both oral IP CBD solution groups received dosing on days 1 and 14, with day 14 administration timed to coincide with peak seizure severity.

The CBD IP group showed significant anticonvulsant efficacy on day 14. On day 1, seizure severity in the vehicle group was low, and no significant treatment effect was observed. However, on day 14, when seizures were fully established, the CBD IP group exhibited a significant reduction in seizure scores. In contrast, the oral CBD group showed a non-significant trend toward reduced seizure severity. This limited efficacy may be attributed to the timing of administration: seizure scores were assessed 1 hour after dosing, whereas the reported Cmax of oral CBD occurs at approximately 8 h post-dose. Thus, CBD plasma levels at the time of assessment may have been subtherapeutic. In comparison, IP administration reaches peak levels within 1–2 h (Uttl et al., 2021; Deiana et al., 2012), potentially providing higher effective concentrations during the evaluation window. Although no CBD solution was administered on day 21, the oral group showed a slight residual effect on severity of seizure. This is likely due to the smaller group size (n = 5 for the oral group, compared with n = 10 for other groups) and greater variability, which could contribute to deviations from the control group response. By this time, the drug is expected to be largely eliminated, so the observed difference is likely attributable to sampling variability rather than a true pharmacological effect.

The IVL5005 demonstrated longer-lasting anticonvulsant efficacy compared to the CBD solution. Both low- and high-dose groups significantly reduced seizure scores on day 14, comparable to the CBD IP group. Notably, the high-dose IVL5005 group sustained efficacy through day 21, with reduced efficacy by day 28; however, the mean seizure score remained lower than that of the vehicle group. Both the CBD solution and high-dose IVL5005 were administered at an equivalent dose of 60 mg. The prolonged efficacy of the LAI formulation aligns with its sustained PK profile, suggesting improved bioavailability relative to the oral CBD solution.

The time-dependent anticonvulsant efficacy observed with IVL5005-60, particularly on days 14 and 21, may be explained by both the characteristics of the PTZ-induced convulsion model and the pharmacological mechanisms of CBD. In the early phase of PTZ administration, neuronal excitability and seizure severity are relatively low, which limits the ability to detect significant treatment effects. With repeated PTZ administration, progressive impairment of GABAergic inhibition and increased excitatory drive result in a lowered seizure threshold and more robust seizure activity after approximately 2 weeks. This pathophysiological context enhances the detectability of pharmacological interventions. CBD is known to exert multiple mechanisms of action, including modulation of excitatory neurotransmission and anti-inflammatory effects (Reddy, 2023). These mechanisms are particularly relevant under conditions of heightened neuronal excitability and neuroinflammation, which become more pronounced as seizure activity progresses. Accordingly, the sustained plasma concentrations of CBD achieved with IVL5005 are more likely to produce measurable anticonvulsant efficacy during the mid-phase of the study, while declining drug exposure by day 28 may have contributed to the reduced efficacy observed at that time.

The exact mechanism of CBD’s anticonvulsant efficacy remain incompletely understood; however, several preclinical studies suggest the CBD acts via multimodal pathways, including non-cannabinoid mechanisms (Scuderi et al., 2009; Reddy, 2023; Fernández-Ruiz et al., 2013; Santos and Fields, 2021). Its anti-convulsant efficacy are mediated through both neuronal and non-neuronal (e.g., microglial) networks, contributing to the regulation of excitability and neuroinflammation. To evaluate these effects, neuronal survival and microglial activation were assessed via histopathological analysis of brain tissue collected at necropsy on day 28.

In the vehicle control group, numerous degenerative cells were observed, with the lowest number of Nissl-positive cells among all groups. Similarly, both the IP and oral CBD solution groups showed comparable levels of neuronal loss. In contrast, the IVL5005 groups exhibited significantly higher number of intact Nissl-positive neurons in both low- and high-dose groups. This preservation of neuronal integrity correlated with lower seizure scores on day 28. Although anticonvulsant efficacy declined by the end of the study, therapeutic effects were still evident in the target tissue.

Neuroinflammation, a key component of epileptogenesis, was assessed by measuring microglial cell activity, a well-established marker of neuroinflammatory response (Putra et al., 2020; Lapato et al., 2017). While the CBD solution groups showed a modest decrease in microglial activity, the IVL5005 exhibited significantly suppressed microglial activation in high-dose group. These findings suggest that the sustained anticonvulsant efficacy of the LAI formulation may be associated with reduced neuroinflammation and preservation of neuronal integrity, as observed at the final necropsy on day 28. In contrast, the CBD solution groups did not show notable histopathological improvement at this time, indicating that their anti-inflammatory and neuroprotective effects may be transient and limited to the immediate post-dosing period.

In this study, the anticonvulsant efficacy of CBD were evaluated using a PTZ-induced convulsion model in adult rats. Rats treated with IVL5005, a CBD-loaded long-acting injectable formulation, exhibited prolonged antiseizure efficacy consistent with the sustained plasma concentrations observed in pharmacokinetic studies. The anticonvulsant efficacy of CBD are believed to involve multiple mechanisms, including modulation of intracellular calcium levels, reduction of neuronal hyperexcitability, and anti-inflammatory and antioxidant actions (Kozela et al., 2010; Arzimanoglou et al., 2020). These mechanisms likely contributed to the observed reduction in seizure severity in IVL5005 treated rats, demonstrating the suitability of the PTZ model for evaluating its anticonvulsant potential.

The poor oral bioavailability of cannabidiol (CBD) is primarily attributed to extensive first-pass metabolism. Following oral administration, CBD undergoes hepatic conversion to metabolites such as 7-hydroxy-CBD (7-OH-CBD) and 7-carboxy-CBD (7-COOH-CBD) (EMA, 2019). Among these, 7-OH-CBD is pharmacologically active and accounts for more than 50% of total plasma exposure. In contrast, 7-COOH-CBD is considered inactive but exhibits exposure levels up to 50-fold higher than the parent compound, contributing to low bioavailability and potential hepatotoxicity (EMA, 2019).

Clinical trials of Epidiolex^®^, an FDA- and EMA-approved oral CBD solution, have reported elevated liver enzyme levels in 5%–20% of patients with epilepsy, with some subjects withdrawn from treatment due to aminotransferase elevations exceeding three times the upper limit of normal (Abu-Sawwa et al., 2020). As a result, dose adjustment and slow titration are recommended in patients with moderate-to-severe hepatic impairment.

In our rat TK study, subcutaneous administration of IVL5005 resulted in a reduced formation of the inactive metabolite 7-COOH-CBD, with a parent-to-metabolite AUC ratio of 31%. By contrast, oral CBD administration in a reference study (Schwotzer et al., 2023) yielded a parent-to-metabolite ratio of 106%, indicating a substantially higher extent of metabolism. These findings suggest that subcutaneous administration of IVL5005 reduces metabolic conversion and may mitigate hepatotoxic risk associated with excessive metabolite exposure.

In the *in vivo* toxicity study, systemic toxicity was evaluated following administration of either oral CBD solution or IVL5005 at pharmacologically effective doses. Consistent with previously reported adverse effects of oral CBD (Gingrich et al., 2023), liver hypertrophy and adrenal vacuolation were observed in the oral solution group. In contrast, no such findings were detected in any animals treated with IVL5005. Given that hepatic effects are closely associated with the extensive metabolism of CBD, these results suggest that IVL5005 could potentially reduce the risk of systemic toxicity by minimizing metabolic burden through a non-oral route of administration, although confirmation in large animal or clinical studies is required. It should also be noted that the microspheres are composed of biodegradable polymers such as PLGA and PLA. These polymers gradually degrade *in vivo* into lactic acid and glycolic acid, which enter normal metabolic pathways and are ultimately eliminated as carbon dioxide and water. Thus, the microspheres do not persist in the body but are progressively degraded and cleared following drug release (Anderson and Shive, 2012).

Furthermore, the IVL-Drug Fluidic^®^ platform has already been applied to other pipeline candidates, for which manufacturing studies were successfully completed and clinical trials are currently ongoing. IVL5005 has also advanced to the scale-up stage in preparation for clinical entry. Based on these prior experiences and established know-how, the production of microspheres with reproducible characteristics using this technology is considered highly feasible.

The primary intended indication for IVL5005 aligns with that of the approved oral CBD solution, Epidiolex^®^, namely, the treatment of rare epilepsies such as LGS and DS. While CBD has minimal psychoactive effects compared with THC, it was initially approved for these rare diseases due to its origin from cannabis. As a CBD-containing long-acting injectable formulation, IVL5005 is therefore also considered primarily for patients with LGS and DS. Clinical use of Epidiolex in these populations has demonstrated sufficient efficacy, and maintaining a stable plasma concentration of CBD is expected to produce comparable anticonvulsant efficacy.

In addition, CBD has been evaluated in several well-established anticonvulsion models, further supporting its applicability to epilepsy indications. Since the present study was conducted in adult rats, future investigations in neonatal or juvenile models may be warranted to better reflect the pediatric populations affected by LGS and DS.

Nevertheless, this study has several limitations. Although the anticonvulsant efficacy of CBD has been demonstrated in preclinical and clinical studies, advancement of IVL5005 into clinical trials will require comprehensive safety evaluation, including studies in large animals such as beagle dogs, with particular attention to potential injection site reactions associated with subcutaneous or intramuscular administration. While the present findings provide valuable insights into the sustained anticonvulsant effects of IVL5005, further studies, including human PK simulations and dose optimization, will be necessary to predict therapeutic plasma concentrations and dosing regimens in patients. Although direct extrapolation of these preclinical findings to humans is not yet possible, the pharmacokinetic data obtained in rats and dogs provide a foundation for future translational studies and will inform the selection of doses and dosing intervals to maintain therapeutic plasma concentrations in humans.

In addition, the PTZ-induced convulsion model used in this study does not replicate the pathophysiology of LGS or DS, and the molecular targets of CBD may differ between epilepsy types. Therefore, caution should be exercised when extrapolating these results to LGS and DS patients, and additional disease-relevant preclinical models are warranted. Potential sources of bias include relatively small sample sizes in some treatment groups and variability in seizure induction and individual responses, which may affect the robustness and precision of the observed results. Future studies using larger cohorts and refined experimental designs will help improve translational relevance and strengthen the predictive value of IVL5005 for clinical application.

In conclusion, we successfully developed a long-acting injectable (LAI) formulation of cannabidiol (CBD) using microfluidic technology. Although the anticonvulsant, anti-neuroinflammatory, and neuroprotective properties of CBD have been demonstrated in both preclinical and clinical studies, currently available oral formulations are limited by low bioavailability, extensive first-pass metabolism, and challenges in patient compliance. In this study, IVL5005, a CBD-loaded LAI, achieved sustained systemic exposure from a single subcutaneous injection and exhibited measurable anticonvulsant efficacy on days 14 and 21 of the PTZ-induced convulsion paradigm, whereas the CBD solution group showed only a transient effect despite receiving the same dose. These findings suggest that CBD LAI formulations may have potential as a long-acting treatment option for epilepsy, although further studies are needed to confirm efficacy across different doses and time points.

## Data Availability

The original contributions presented in the study are included in the article/Supplementary Material, further inquiries can be directed to the corresponding authors.

## Abbreviations

CBD, cannabidiol; DS, dravet syndrome; EE, encapsulation efficiency; IBA1, ionized calcium-binding adaptor molecule 1; IP, intraperitoneal injection; LAI, long-acting injectable; LGS, lennox–gastaut syndrome; PK, pharmacokinetics; PLGA, poly lactic-co-glycolic acid; PO, oral administration; PTZ, pentylenetetrazole; SEM, scanning electron microscopy; SC, subcutaneous.
