# Supplementary material for: Anti-convulsant efficacy of long-acting injectable cannabidiol formulation (IVL5005) in the pentylenetetrazol-induced convulsions, with pharmacokinetic characterization
Source: Front Pharmacol. 2025 Oct 23;16:1692123. doi: 10.3389/fphar.2025.1692123 (PMC12589995; doi:10.3389/fphar.2025.1692123)
Supplement: Supplementary file 1 [file DataSheet1.pdf]

Formulation preparation

Microspheres containing CBD were produced by injecting biodegradable polymer solutions and aqueous solutions into microchannels using the Elveflow OB1 MK3+ microfluidic flow controller. Monodispersed droplets were formed due to the interaction of the polymer and aqueous phases under controlled flow (Supplement Figure 1).

Supplement Fig 1. Manufacturing and characterization of CBD-loaded microspheres.

a

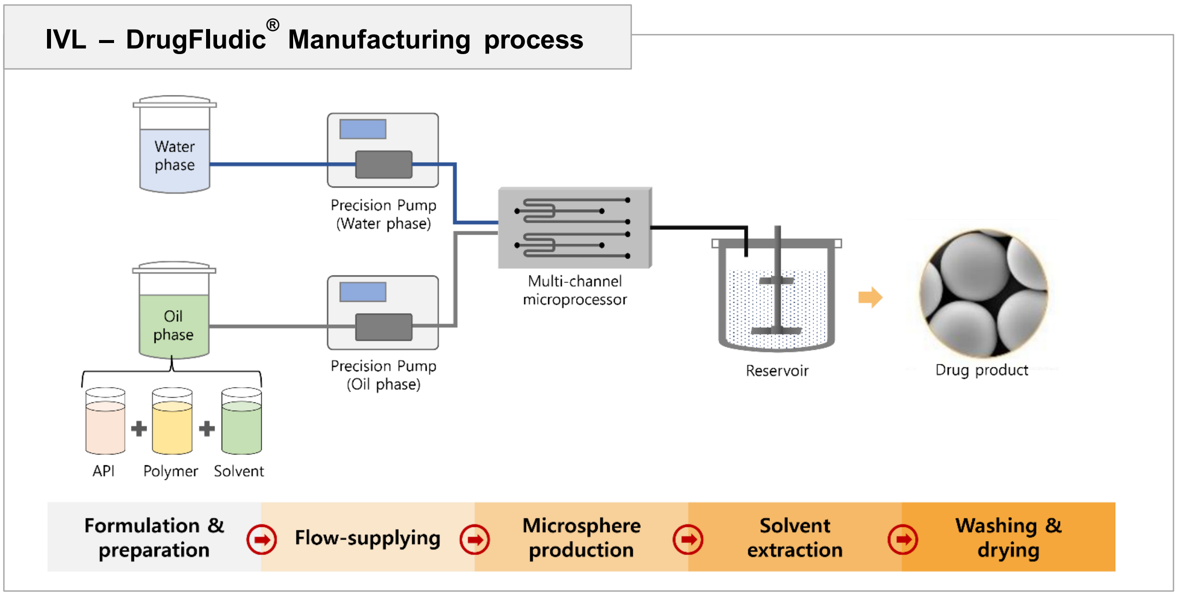

Schematic illustrating the microsphere manufacturing process using microfluidic technology (IVL-DrugFluidic®). Monodispersed droplets were formed due to the interaction of the polymer and aqueous phases under controlled flow.

Animal information

Animals for pharmacokinetic and efficacy studies

Rats were selected for the pharmacokinetic study because repeated blood sampling from the same animal is feasible, allowing detailed evaluation of plasma drug concentrations after LAI administration. Using the same species as in the efficacy studies also enables direct correlation of pharmacokinetic and pharmacodynamic outcomes. Beagle dogs were included as a non-rodent species because they are widely accepted in drug development for PK studies and provide translational data relevant to humans. Additionally, since the LAI formulation is a development based on the previously studied oral CBD formulation (Epidiolex®), the same species (rat and dog) were used to enable direct comparison with prior PK data and facilitate translation of findings across formulations.

Specific pathogen-free male Sprague-Dawley (SD) rats were used for the pharmacokinetic (PK) and efficacy studies. For the original PK study evaluating five IVL5005 formulations, 25 rats were used, and for the efficacy study, 45 rats were used (Koatech, Pyeongtaek, Republic of Korea). Additionally, a separate PK study comparing IVL5005 with oral CBD solution was conducted using 30 SD rats. The breeding room conditions were maintained as follows: temperature 20–26°C (original studies) or 22 ± 3°C (additional PK study), relative humidity 40–70% (original studies) or 50 ± 20% (additional PK study), and illuminance 150–300 Lux. The studies were approved by the Institutional Animal Care and Use Committee (IACUC) of Jeonbuk National University Hospital (JBNUH-IACUC-2023-10 and JBNUH-IACUC-2023-20 for the original studies) and IACUC of Croen Inc (approval no. 23R1116 for the additional PK study).

6 dogs were used for PK study. The breeding room conditions were maintained as follows: temperature at 18–24°C, relative humidity at 30–70%, and illuminance of 150–300 Lux. The study was approved by Institutional Animal Care and Use Committee (IACUC) of Biotoxtech Co., Ltd. (IACUC-241000000040).

In the rat study, animals were euthanized at the end of the experiment. Humane endpoints were established according to standard institutional guidelines to minimize suffering, and no early euthanasia was required during the study. In the dog study, animals were returned to the facility's holding population following study completion. After study completion, the dogs were transferred to the facility's holding population and cared for according to standard operating procedures. As per these procedures, clinical signs were monitored once daily, and body weight was measured every 4 weeks.

Throughout the study, all animals were managed in accordance with the test facility's standard operating procedures to minimize pain, suffering, and distress. Environmental enrichment was provided continuously by supplying toys. Additionally, the housing environment was maintained by cleaning the cages at least once daily and continuously monitoring and controlling temperature and humidity.

The sample size for each experimental group was determined based on commonly accepted practices in preclinical pharmacokinetic and efficacy studies. No formal a priori statistical sample size calculation was performed; however, the selected number of animals per group (e.g., 5–10 rats for treatment groups, 3-5 rats for control) was considered sufficient to detect meaningful differences and observe outcomes while minimizing animal use. All animals included in the study were healthy at the start of the experiment and were monitored throughout according to the study protocol. No animals or data points were excluded from the analysis, and all observations were included in the final dataset. In the PTZ efficacy study, three surplus rats remained after group allocation, and these animals were subsequently utilized in a pilot study to determine the dosing interval for repeated PTZ administration. This approach minimized the need for additional animals and was conducted under the oversight of the IACUC to ensure compliance with animal welfare standards.

The exact number of animals (n) used for each analysis in each experimental group is summarized in the table below.

Supplement Table 1. Animal information

Study	Animal no.	Age	Weight	Breed	Sex
Rat PK (five IVL5005 formulations)	25 rats<br>: 5 groups, 5 rats/group	6 weeks at initiation dosing	190 g – 330 g (from dosing initiation to study completion)	Sprague-Dawley rat	Male
Rat PK (CBD sol. vs IVL5005)	30 rats<br>: 2 groups, 15 rats/group	6 weeks at initiation dosing	218.6 g at initiation dosing	Sprague-Dawley rat	Male
Dog PK	6 dogs<br>: 1 group, 6 dogs/group	15 months at initiation dosing	10.9 kg – 10.4 kg (from dosing initiation to study completion)	Beagle dog	Male
Rat efficacy (PTZ model)	45 rats<br>: G1 (control) 10 rats/group<br>: G2 (CBD solution I.P. injection) 10 rats/group<br>: G3 (CBD solution oral administration) 5 rats/group<br>: G4 (IVL5005 30 mg) 10 rats/group<br>: G5 (IVL5005 60 mg) 10 rats/group	7 weeks at initiation dosing	184.8 g – 315.5 g (from dosing initiation to study completion)	Sprague-Dawley rat	Male
Rat toxicity + Metabolite analysis	36 rats<br>: G1 (control) 3 rats/group<br>: G2 (CBD solution oral administration) 9 rats/group<br>: G3 (IVL5005 30) 9 rats/group<br>: G4 (IVL5005 60) 9 rats/group<br>: Metabolite analysis group 6 rats/group	6 weeks at initiation dosing	215.9 g – 442.4 g (from dosing initiation to study completion)	Sprague-Dawley rat	Male

The doses for rats and dogs were calculated based on typical average body weights commonly used in preclinical studies (approximately 0.3 kg per rat and 10 kg per dog), rather than the actual measured body weights of the animals in this study (James Edgar Mccutcheon et al., 2009, So-Young Choi et al., 2011).

In vivo rat PK, formulation screening

5 IVL5005 formulations were selected and rat PK study was conducted.

Supplement Table 2. IVL5005 rat screening study, PK parameters

**Test no.**	**Dose (mg/head)**	**T_{max} (h)**	**C_{max} (ng mL^{-1})**	**$AUCl_{ast}$ ng×h/mL**	**AUC0-24 ng×h/mL**	**AUC0-24/AUClast ratio**
#5	30	8	70.12 ± 37.77	10237	1228	12.00
#6	30	4	59.22 ± 22.06	8389	1075	12.81
#7	30	8	41.31 ± 10.88	9682	813	8.40
#8	30	8	102.53 ± 48.47	14857	1351	9.09
#9	30	8	68.98 ± 12.37	8741	1156	13.23

PTZ-induced seizure model, pilot study

A pilot study was conducted using three male Sprague-Dawley rats to evaluate the induction and stability of seizure behavior following repeated PTZ administration. PTZ was administered intraperitoneally at 37.5 mg/kg according to the following dosing schedule: days 1, 3, 5, 7, 12, 21, 28, and 35. After each administration, seizure grade was assessed for 30 minutes. Seizure scores were monitored for five weeks after the initiation of dosing. The results demonstrated that stable seizure grades were maintained from the second week onward.

Supplement Fig 2. Seizure induction in PTZ-induced seizure model, pilot study

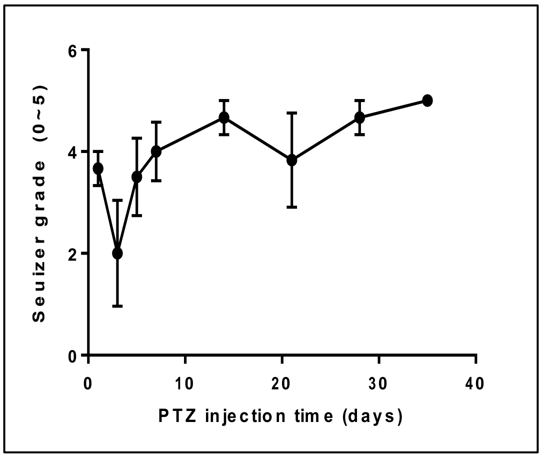

Histopathological analysis in PTZ-induced rat seizure model

Brain tissues were subjected to Nissl staining and IBA-1 immunostaining. The mean ± standard error values for the number of Nissl-positive and IBA-1-positive cells in each group are presented, along with the corresponding p-values.

Supplement Table 3. Histopoatholotical analysis in PTZ-induced rat seizure model

Group	Nissl staining			IBA immunostaining
	CA1	CA3	DG	Hippocampus
G1. Vehicle control	126.3±6.8	132.5±16.2	280.3±26.2	100.00±20.68
G2. CBD-IP (60 mg)	124.5±7.9	130.3±12.2	285.0±19.0	78.81±19.18
G3. CBD-PO (60 mg)	128.3±7.2	124.7±10.9	293.7±12.6	67.07±37.76
G4. IVL5005 (30 mg)	134.7±6.7*	142.5±9.1	304.6±25.0	66.99±12.96
G5. IVL5005 (60 mg)	133.9±7.6*	148.3±10.1*	306.7±12.9	50.01±15.45*

Quantitative analysis of Nissl staining cell bodies in hippocampal CA1, CA3, and DG region, and quantitative analysis of positive IBA1 positive signal intensity in hippocampus. All data were expresed as mean ± SD. *p<0.05 vs. vehicle control.

Animal toxicity study

The objective of this study was to evaluate the potential toxicity of IVL5005 following subcutaneous injection in Sprague-Dawley rats. In addition, a comparative group receiving CBD oral solution was included to assess differences in adverse effects relative to the test item group.

Supplement Table 4. Toxicity study design

Group	Route of administration	Dose level (mg/animal)	Dose volume (ml/animal)	Number of animals
Group 1. Vehicle control	SC	0	1.5	3
Group 2. CBD solution	PO	60	1.5	9
Group 3. IVL5005 Low	SC	30	1.5	9
Group 4. IVL5005 High	SC	60	1.5	9

Evaluations were based on mortality, clinical signs, body weight, hematology and clinical chemistry, organ weights, and histopathological examinations.

Clinical observations

All animals were observed daily for abnormal clinical signs or confirmation of normal status, and morbidity and mortality were monitored twice daily. No systemic findings were observed during the study, and all clinical findings were considered tolerable and not treatment-related.

Supplement Table 5. Summary of clinical signs

Clinical signs From Day 1 (Start Date) to 28	Group 1	Group 2	Group 3	Group 4
Loss of fur, face, right	-	D19 to D28	-	-
Injection site, Nodule	-	-	D10 to D28	D10 to D28

Group 1: Vehicle control, Group 2: CBD oral solution (60 mg), Group 3: IVL5005 Low (30 mg), Group 4: IVL5005 High (60 mg)

Body weight measurements

Body weight of all animals was recorded once weekly throughout the study. No treatment-related changes in body weight were observed compared with the vehicle control group.

Supplement Table 6. Summary of body weights

Group		Days relative to start date				
		D1 (g)	D7 (g)	D14 (g)	D21 (g)	D28 (g)
Group 1	Mean	217.4	277.5	345.5	393.5	451.1
	SD	1.7	3.7	9.6	8.9	16.6
Group 2	Mean	216.4	271.6	334.8	380.0	425.9
	SD	11.3	18.0	27.8	35.6	42.1
Group 3	Mean	215.5	270.6	332.8	379.0	425.2
	SD	7.5	9.6	12.6	16.8	18.0
Group 4	Mean	216.6	272.6	339.3	386.1	440.3

	SD	12.6	19.6	29.7	36.9	47.3

Group 1: Vehicle control, Group 2: CBD oral solution (60 mg), Group 3: IVL5005 Low (30 mg), Group 4: IVL5005 High (60 mg)

Clinical pathology

At necropsy, blood samples were collected for hematology and clinical chemistry analyses. All variations in the hematology parameters, regardless of statistical significance, were sporadic; within biological variation in this species; small magnitude; and considered not to be related to test or comparative items dosing.

Supplement Table 7. Summary of Hematological parameter

Group		RBC (10^6/μL)	HGB (g/dL)	HCT (%)	MCV (fL)	MCH (pg)	MCHC (g/dL)	PLT (10^3/μL)	WBC (10^3/μL)
Group 1	Mean	6.99	14.4	41.0	58.6	20.6	35.1	1020	9.41
	SD	0.15	0.4	1.6	1.4	0.4	0.3	17	1.33
Group 2	Mean	6.99	14.4	41.5	59.4	20.6	34.7	979	10.58
	SD	0.29	0.6	1.9	1.1	0.4	0.5	163	1.37
Group 3	Mean	7.18	14.5	42.0	58.6	20.2	34.5	1037	8.37
	SD	0.41	0.3	1.5	1.5	0.8	0.6	63	1.47
Group 4	Mean	7.05	14.4	41.5	58.9	20.4	34.7	1024	10.15
	SD	0.19	0.1	0.9	1.4	0.5	0.5	78	1.81

Group 1: Vehicle control, Group 2: CBD oral solution (60 mg), Group 3: IVL5005 Low (30 mg), Group 4: IVL5005 High (60 mg)

Supplement Table 8. Summary of Clinical chemistry parameter

Group		ALT (U/L)	AST (U/L)	ALP (U/L)	BUN (mg/dL)	Crea (mg/dL)	TP (g/dL)	Alb (g/dL)	A/G ratio
Group 1	Mean	30.0	75.5	420.3	14.6	0.45	5.6	2.3	0.68
	SD	5.6	13.5	9.0	4.4	0.06	0.2	0.1	0.06
Group 2	Mean	23.0*	85.6	533.6	11.9	0.43	5.7	2.3	0.70
	SD	2.9	13.5	58.1	1.8	0.03	0.2	0.1	0.04
Group 3	Mean	30.4	93.6	544.9	13.7	0.42	5.6	2.3	0.70
	SD	4.5	15.7	112.6	1.5	0.01	0.2	0.1	0.03
Group 4	Mean	29.1	89.7	563.6	12.8	0.41	5.8	2.3	0.69
	SD	4.7	8.5	92.5	1.2	0.04	0.3	0.1	0.05

Continued

Group		T-Chol (mg/dL)	TG (mg/dL)	Glu (mg/dL)
Group 1	Mean	71	58	112
	SD	14	14	3
Group 2	Mean	67	85	109
	SD	11	30	12
Group 3	Mean	68	42	113
	SD	5	10	10
	Mean	70	51	123

Group 4	SD	12	24	13

Group 1: Vehicle control, Group 2: CBD oral solution (60 mg), Group 3: IVL5005 Low (30 mg), Group 4: IVL5005 High (60 mg) / Anova&Dunnett, *=$p<0.05$

Postmortem evaluation

At necropsy, representative organ weights were recorded, and microscopic examination of fixed tissues was performed. The following organs were collected, fixed, and processed into slides for histopathological evaluation:

Supplement Table 9. Summary of postmortem evaluation

Organ/Tissue	Organ Weight	Collected and Preserved	Histopathology Evaluation (H&E staining)
BRAIN	Yes	Yes	Yes
EPIDIDYMIS	-	Yes	-
GLAND, ADRENAL	-	Yes	Yes
GLAND, PARATHYROID a)	-	Yes	-
GLAND, THYROID	-	Yes	Yes
HEART	Yes	Yes	-
KIDNEY	Yes	Yes	Yes
LARGE INTESTINE, CECUM	-	Yes	-
LARGE INTESTINE, COLON	-	Yes	-
LARGE INTESTINE, RECTUM	-	Yes	-
LIVER	Yes	Yes	Yes
LUNG/BRONCHUS	-	Yes	-
PANCREAS	-	Yes	-
PEYER'S PATCH	-	Yes	-
SMALL INTESTINE, DUODENUM	-	Yes	-
SMALL INTESTINE, ILEUM	-	Yes	-
SMALL INTESTINE, JEJUNUM	-	Yes	-
SPLEEN	Yes	Yes	Yes
STOMACH	-	Yes	-
TESTIS	-	Yes	-
THYMUS	-	Yes	-

Yes = Procedure to be conducted, - = Not applicable

Organ weight

There were no treatment-related changes were observed. All variations in the organ weights and organ weight ratio, regardless of statistical significance, showed no dose-response; were within normal biological variation of this species; were small in magnitude; lacked correlation

with other relevant parameter, and considered not to be related to test or comparative items dosing.

Supplement Table 10. Summary of Organ weights

Group		Terminal body weight (g)	Brain (g)	Relative brain (%)	Heart (g)	Relative heart (%)	Kidney (g)	Relative kidney (%)
Group 1	Mean	418.4	2.0775	0.4969	1.3275	0.3179	2.8983	0.6935
	SD	13.5	0.1019	0.0301	0.0888	0.0315	0.1270	0.0454
Group 2	Mean	399.2	2.0205	0.5100	1.2546	0.3152	2.9538	0.7405
	SD	38.9	0.1320	0.0562	0.0923	0.0165	0.3811	0.0229
Group 3	Mean	398.4	2.0399	0.5125	1.2944	0.3250	2.7245	0.6841
	SD	18.4	0.0806	0.0200	0.0604	0.0077	0.1564	0.0323
Group 4	Mean	412.0	2.1238	0.5195	1.3720	0.3335	2.8942	0.7018
	SD	45.1	0.1162	0.0496	0.1468	0.0197	0.3886	0.0427

Continued

Group		Liver (g)	Relative liver (%)	Spleen (g)	Relative spleen (%)
Group 1	Mean	12.1758	2.9112	0.7680	0.1835
	SD	0.1324	0.0641	0.0277	0.0024
Group 2	Mean	13.7988	3.4448	0.8051	0.2006
	SD	2.0926	0.2324	0.1622	0.0278
Group 3	Mean	11.5736	2.9042	0.7987	0.2004
	SD	0.7375	0.1036	0.0782	0.0157
Group 4	Mean	12.3592	2.9819	0.9126	0.2209*
	SD	2.2471	0.2851	0.1345	0.0149

Group 1: Vehicle control, Group 2: CBD oral solution (60 mg), Group 3: IVL5005 Low (30 mg), Group 4: IVL5005 High (60 mg) / Anova&Dunnett, *=p<0.05

Microscopic findings

IVL5005 and CBD oral solution-related histopathological findings were observed at the liver and adrenal gland.

Centrilobular hepatocellular hypertrophy in the liver and increased vacuolation in the zona fasciculata in the adrenal gland were observed in the CBD solution oral dose group.

Supplement Table 11. The incidence and sevierty of the remarkable microscopic findings

Microscopic findings	G1	G2	G3	G4
Adrenal gland				
Examined	3	9	9	9
Vacuoloation; cortex; zona fasciculata; increased, diffuse	-	5	-	-
Grade 1	-	2	-	-
Grade 2	-	3	-	-
Liver				
Examined	3	9	9	9
Hypertrophy; centrilobular; hepatocyte	-	4	-	-

Grade 1	-	4	-	-

Group 1: Vehicle control, Group 2: CBD oral solution (60 mg), Group 3: IVL5005 Low (30 mg), Group 4: IVL5005 High (60 mg)

Supplement Fig 3. Representative microscopic findings

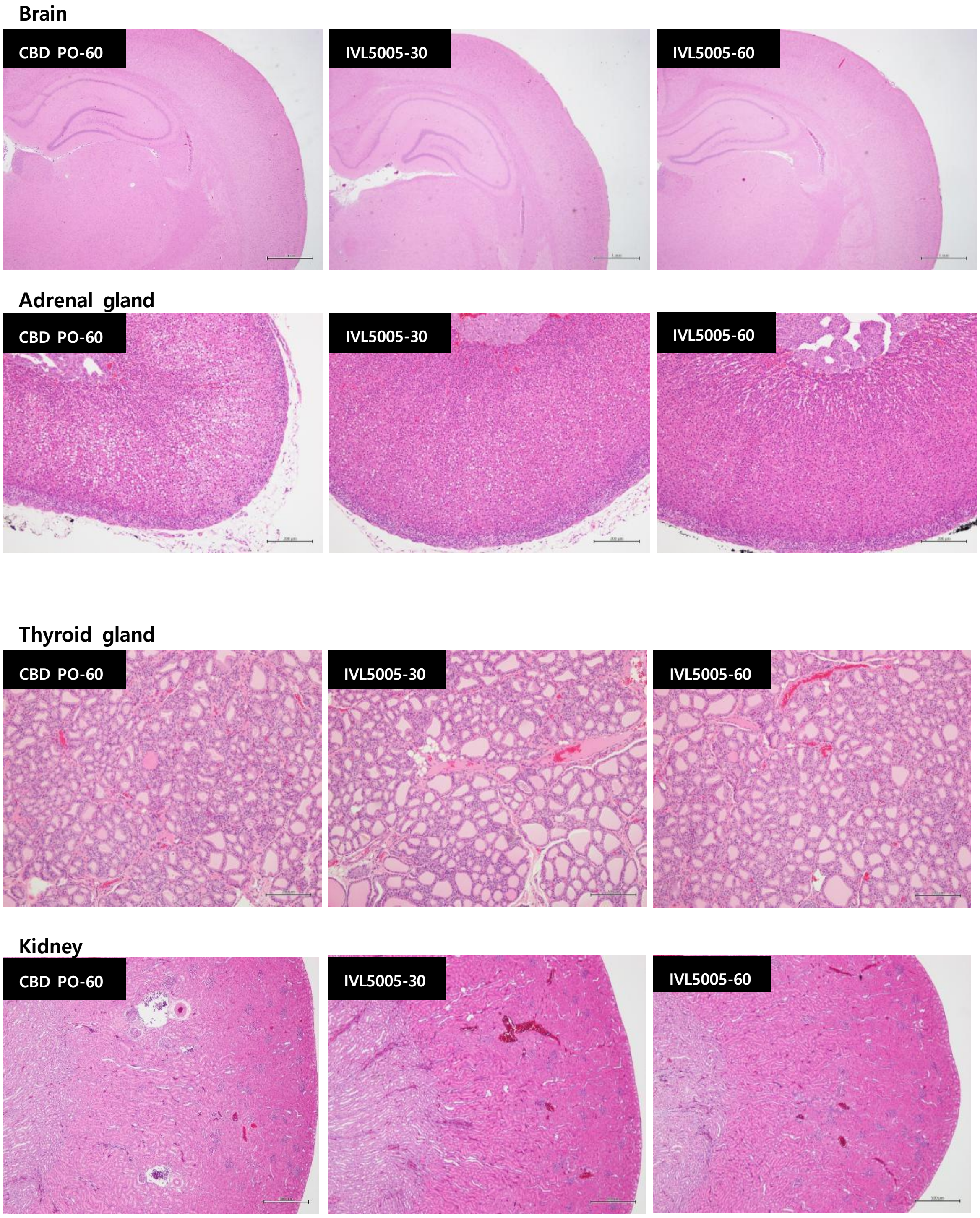

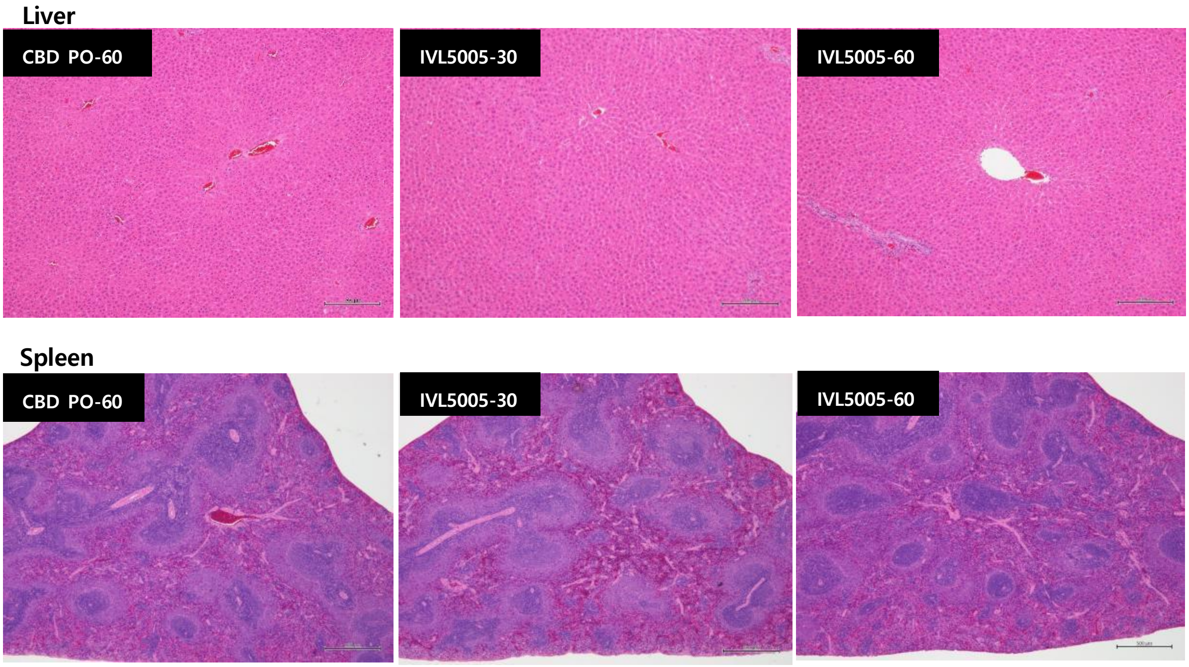
Liver
CBD PO-60
IVL5005-30
IVL5005-60
Spleen
CBD PO-60
IVL5005-30
IVL5005-60
